# Fenchone Ameliorates Constipation-Predominant Irritable Bowel Syndrome via Modulation of SCF/c-Kit Pathway and Gut Microbiota

**DOI:** 10.4014/jmb.2308.08011

**Published:** 2023-10-28

**Authors:** Li Cui, Bin Zhang, Shuting Zou, Jing Liu, Pingrong Wang, Hui Li, Zhenhai Zhang

**Affiliations:** 1 Affiliated Hospital of Integrated Traditional Chinese and Western Medicine, Nanjing University of Chinese Medicine, Nanjing 210028, P.R. China; 2 Digestive Department, Nanjing Lishui District Hospital of Traditional Chinese Medicine, Nanjing 211200, Jiangsu, P.R. China; 3 Jinling Clinical Medical College, Nanjing University of Chinese Medicine, Nanjing 210016, P.R. China

**Keywords:** IBS-C, fenchone, SCF/c-Kit pathway, gut microbiota

## Abstract

In this study we sought to elucidate the therapeutic effects of fenchone on constipation-predominant irritable bowel syndrome (IBS-C) and the underlying mechanisms. An IBS-C model was established in rats by administration of ice water by gavage for 14 days. Fenchone increased the reduced body weight, number of fecal pellets, fecal moisture, and intestinal transit rate, and decreased the enhanced visceral hypersensitivity in the rat model of IBS-C. In addition, fenchone increased the serum content of excitatory neurotransmitters and decreased the serum content of inhibitory neurotransmitters in the IBS-C rat model. Meanwhile, western blot and immunofluorescence experiments indicated that fenchone increased the expressions of SCF and c-Kit. Furthermore, compared with the IBS-C model group, fenchone increased the relative abundance of *Lactobacillus*, *Blautia*, *Allobaculum*, *Subdoligranulum*, and *Ruminococcaceae_UCG-008*, and reduced the relative abundance of *Bacteroides*, *Enterococcus*, *Alistipes*, and *Escherichia-Shigella* on the genus level. Overall, fenchone ameliorates IBS-C via modulation of the SCF/c-Kit pathway and gut microbiota, and could therefore serve as a novel drug candidate against IBS-C.

## Introduction

Irritable bowel syndrome (IBS) is a common functional gastrointestinal (GI) disorder, with features of abdominal pain or discomfort and intestinal dysmotility together with mental symptoms of depression and anxiety [1]. IBS affects around 12% of the world’s population, and the prevalence rates in the West and around Asia are 9–22% and 4–10%, respectively [2]. The pathogenesis of IBS is closely associated with GI motility disorder, visceral hypersensitivity, imbalanced gut microbiota, and immunity damage. According to the Rome IV criteria, IBS can be divided into four different subtypes according to bowel habits and stool morphology: constipation-predominant IBS (IBS-C), diarrhea-predominant IBS (IBS-D), mixed IBS (IBS-M), and unclassified IBS (IBS-U)[3]. Since IBS-C accounts for nearly one-third of all IBS patients, it receives the most attention. This chronic GI disease is denoted by abdominal pain, cramping, and difficulty in evacuating stools. IBS-C is usually influenced by environmental factors as well as intestinal flora disturbance, GI hypomotility, and epithelial barrier dysfunction. The current treatments for IBS-C patients include changes in lifestyle, over-the-counter laxatives, and analgesics [4]. However, the majority of IBS-C patients still suffer persistent symptoms despite active treatment [5]. Until now, no satisfactory treatment for IBS-C has been found. A therapeutic approach that seeks to reduce constipation-related symptoms and restore balance in the intestinal flora may be a novel research strategy. In addition, functional foods as a novel therapy for disease have also attracted wide attention from researchers.

The occurrence and development of IBS-C are associated with intestinal transit rate, aquaporin content, enteric nervous system dysfunction, related neurotransmitter expression levels, and intestinal flora disorders [6-9]. Imbalance of the intestinal flora is closely related to Alzheimer's disease, colitis, IBS-C, and other diseases [10-12]. It has been reported that the composition of intestinal microorganisms in healthy individuals differed from that found in patients, and disturbances of intestinal flora contributed to aberrant gut motility [13]. DuPont *et al*. showed that IBS-C volunteers presented with delayed gastric emptying and prolonged gut transit times [14]. Interstitial cells of Cajal (ICCs) are the main component of the GI tract and are closely correlated with bowel movements [15]. Proto-oncogene coding receptor tyrosine kinase, or c-Kit, is expressed by ICCs, and stem cell factor (SCF) is a ligand of c-Kit. The SCF/c-Kit signaling pathway is crucially important in the proliferation and function of ICCs. ICCs are involved in the pathogenesis of multiple diseases caused by GI peristalsis dysfunction [16]. Thus, regulating ICC in the GI might be a valuable treatment method to boost intestinal movement.

Essential oils are a mixture of natural, volatile, and aromatic compounds obtained from different plants and are responsible for many biological activities [17]. Importantly, essential oils such as *Trachyspermum ammi* L. oil, citrus aurantium essential oil, and peppermint oil are efficacious in relieving symptoms attributable to IBS [18-20]. Fenchone exists as an irregular bicyclic monoterpene ketone and is found in the essential oil of multiple fragrant plants, including *Foeniculum vulgare* and *Peumus boldus* [21]. Pharmacological studies have confirmed that fenchone possesses anti-inflammatory, antioxidant, antidiarrheal, and antifungal activities [22-24]. However, there has been no report of fenchone use in IBS-C therapy. Based on the protective effects of other essential oils against IBS-C, we speculated that fenchone may exert a therapeutic effect in IBS-C. To confirm this hypothesis, an IBS-C rat model was established by cold (0–4°C) intragastric water. We then evaluated the ameliorating effect of fenchone on IBS-C-related symptoms, including body weight, number of fecal pellets, visceral hypersensitivity, and intestinal transit. The SCF/c-Kit pathway and the changes in intestinal flora composition were also explored in the treatment of IBS-C by using fenchone.

## Materials and Methods

### Reagent and Chemicals

Fenchone (≥98.0%, GC) was bought from Aladdin Biochemical Technology Co., Ltd. (Shanghai, China). Linaclotide was purchased from Meckin (China). Tween-80 was obtained from Shandong Deyan Chemical Co., Ltd. (China). Antibodies against SCF, c-Kit, and GAPDH were obtained from Santa Cruz Biotechnology (USA). Rat substance P (SP), motilin (MTL), 5-hydroxytryptamine (5-HT), and vasoactive intestinal peptide (VIP) ELISA kits were purchased from Shanghai Lianke Biotech Co., Ltd. (China).

### Animals

Forty-two SPF male SD rats (220 ± 20 g) were purchased from Nantong University. The experiments were conducted at the Animal Experimental Center of Jiangsu Province Academy of Traditional Chinese Medicine. Animals were fed ad libitum with water and food under standardized condition (25°C; RH: 55 ± 5%; light-dark schedule: lights on 8 a.m. to 8 p.m.). Animal procedures were conducted under the approved guidelines of the Animal Ethics Committee of Jiangsu Provincial Academy of Chinese Medicine (NO.AEWC-20230323-285), and followed the National Research Council Guide.

### IBS-C Model Establishment, Drug Intervention and Sample Collection

Forty-two SD rats were classified into 7 groups equally: normal control group, IBS-C model group, Tween-80 group, linaclotide group (3 mg/kg), low-dose fenchone group (50 mg/kg), medium-dose fenchone group (100 mg/kg), and high-dose fenchone group (200 mg/kg). Fenchone was dissolved in 0.1% Tween 80. To establish the IBS-C rat model, except the normal control group, all other groups were orally treated with 2 ml cold water (0-4°C) once a day for 14 consecutive days [25]. The normal control group received an equal amount of normal saline instead of cold water. To avoid the influence of biorhythm, the gavage was fixed at 9 a.m. After the IBS-C model was established, rats in the Tween-80 group received 0.1% Tween-80 by gastric lavage, whereas those in the linaclotide group and three-different-dose fenchone groups were orally administered with corresponding drug per day from day 15 to day 28. The normal control and IBS-C model groups were treated orally with equal volume normal saline. The animals were provided with adequate feed and water during the experiment. At the end, the fecal pellet number and total wet and dry fecal weights were detected and recorded. The fresh fecal samples were stored in liquid nitrogen for analysis of gut microbiota. Blood samples taken from the orbital sinus were gathered in sterile anticoagulant blood tubes. The serum was prepared with condition of 3000 ×*g*, 4°C for 10 min, and the supernatants were stored at −80°C. Subsequently, the rats were sacrificed via cervical dislocation. Colon tissues were frozen at −80°C for subsequent analysis.

### Detection of Body Weight and Fecal Output

Rats were housed in a metabolic cage. Body weight was supervised weekly. Fecal samples were dried in an oven at 80°C to constant weight. Fecal moisture content was determined by (wet fecal weights − dry fecal weights)/wet fecal weights × 100% (1).

### Measurement of Visceral Hypersensitivity and Intestinal Transit

Visceral hypersensitivity was determined by the abdominal withdrawal reflex (AWR) score. Rats were fasted overnight before the visceral hypersensitivity test began. A 6-F catheter with glycerol lubrication was inserted into the rectum. Adhesive tape was applied to fix the catheter to the tail. Normal saline was introduced to distend the catheter balloon. AWR scores were assigned through visual observation of animal responses: 0 = no response during colorectal dilation; 1 = static during dilation, occasional response during stimulation; 2 = slight contraction of abdominal muscles; 3 = abdominal lift; 4 = body arched, pelvis raised (25).

Rats were fasted overnight before receiving an oral dose of 2 ml suspension of a 10% activated carbon powder to evaluate the GI transit rate. Rats were euthanized 0.5 h after gavage, and the intestine from the pyloric junction to the ileocecal valve was isolated. The GI transit rate was obtained based on the following formula: GI transit rate = the distance advanced by carbon powder/small intestine total length × 100% (26).

### Enzyme-Linked Immunosorbent Assay (ELISA)

The gastrointestinal hormone levels in serum samples, including SP, MTL, 5-HT, and VIP were detected via commercially available ELISA kits. The experiment was conducted according to the manufacturer’s instructions.

### Western Blot Analysis

The colonic tissue was extracted and lysed into tissue homogenate by adding lysate buffer. The total protein in the lysate was separated via SDS-PAGE gel. After electrophoresis, the total protein was transferred to a PVDF membrane and blocked with 5% BSA. Following that, the membrane was incubated with corresponding primary antibody (anti-c-Kit, anti-SCF) at 4°C overnight, and washed in TBST buffer solution. Then, the membrane was incubated with the secondary antibody coupled with horseradish peroxidase at 25°C for 1.5 h, and washed. Protein bands were measured via an ECL detection kit, and GAPDH was applied to estimate the content of each protein.

### Immunofluorescence Test

The colon tissue was fixed in 10% buffered formalin, and cut into 5 μm thickness. The sections were dewaxed in xylene, hydrated, and then treated with citric acid buffers to repair antigens. The primary antibody (anti-c-Kit, anti-SCF) was added to the sections and incubated for 12 h at 4°C. Then, the tissue was washed using PBS thoroughly and incubated with FITC-labeled fluorescent secondary antibody in the dark at 37°C for 90 min. The sections were re-stained with DAPI for 5 min, rinsed with PBS, and sealed with anti-fluorescence quenching tablets. The fluorescent labels were observed via Nikon confocal laser scanning microscope, and the positive expression of colon tissues was determined based on a digital image device (Image-Pro Plus 6.0), and the average absorbance was calculated.

### 16S rRNA Gene Sequencing and Bioinformatics Analysis

Fresh rat feces (150 mg) were collected and analyzed according to a previously published study. The V3-V4 regions of 16S rRNA were amplified via a specific primer (338F 5'-ACTCCTACGGGAGGCAGCA-3'; 806R 5'-GGACTACHVGGGTWTCTAAT-3'). The amplification product contents were measured via agarose gel electrophoresis during the experiment study. The amplified product was purified using the E.Z.N.A. Kit (Omega, USA). Sequencing libraries were obtained based on the NEBNext Ultra II DNA Library Prep Kit. The quality of the library was detected on the Qubit@ 2.0 Fluorometer (Thermo Fisher Scientific, USA). Finally, the Illumina Nova6000 platform was used for sequencing, and a paired reading code of 250 bp was generated (Guangdong Magigene Biotechnology Co., Ltd. China). The quality of the original data was controlled by Fastp 0.14.1 to determine the matching end cleaning label that meets the requirements. The R software was applied to identify the species, composition of community, and richness of species. Alpha diversity was applied in analyzing the complexity of species diversity. Beta diversity analysis was conducted to assess differences of samples.

### Statistical Analysis

Statistical analyses were conducted based on SPSS 26.0 (IBM, USA). Comparisons between two or more groups were drawn through an unpaired *t*-test or ANOVA. All data were expressed as mean ± SD. *p* < 0.05 was taken to judge the significant difference.

## Results

### Effect of Fenchone on IBS-C-Related Indicators

**Fig. 1:**
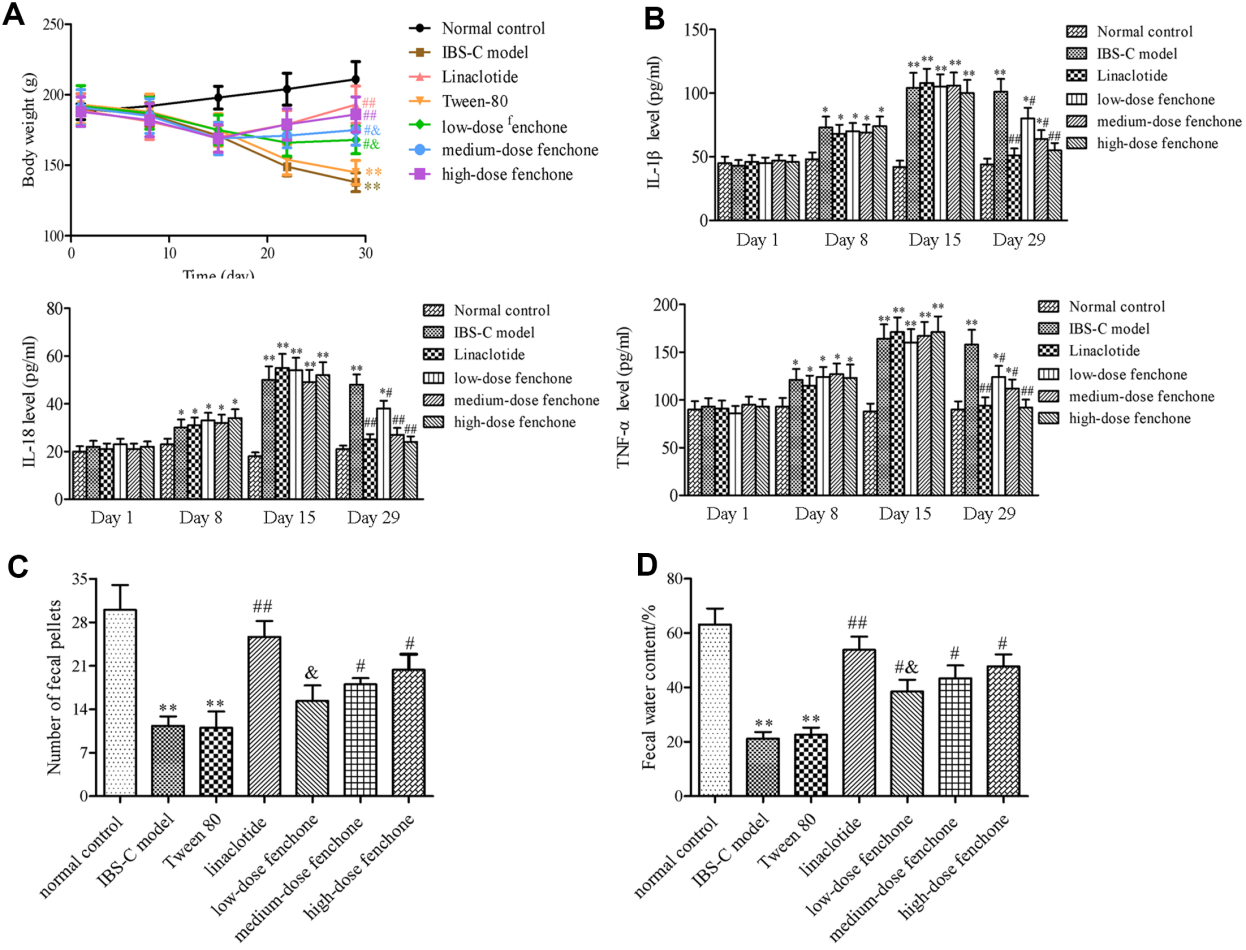
Effect of fenchone on IBS-C-related indicators. (**A**) Body weight. (**B**) inflammatory cytokines in serum. (**C**) number of fecal pellets. (**D**) fecal water content. Data were represented as mean ± SD. *p < 0.05, **p < 0.01, compared with normal control group; ^#^*p* < 0.05, ^##^*p* < 0.01, compared with IBS-C model group; ^&^*p* < 0.05, compared with linaclotide group.

As shown in Fig. 1A, the body weight of IBS-C model rats was obviously decreased compared with that of normal control rats (*p* < 0.01). However, compared with IBS-C model rats, linaclotide and fenchone significantly increased body weight (*p* < 0.05, *p* < 0.01). Among the different-dosages-of-fenchone groups, the body weight in the low- and medium-dose fenchone was lower than that in the linaclotide group (*p* < 0.05). The contents of IL-1β, IL-18, and TNF-α in serum increased with the extension of ice water induction time, and there was significant difference between normal control group and ice water-induced groups (*p* < 0.05, *p* < 0.01). However, the contents of IL-1β, IL-18, and TNF-α in the low-, medium- and high-dose fenchone groups were lower than those in the IBS-C group (*p* < 0.05, *p* < 0.01) (Fig. 1B). The fecal pellet number and fecal water content were determined to assess the defecation activity exerted by fenchone in the IBS-C model. Compared to the normal control group, the fecal pellet number and fecal water content were decreased in the IBS-C and Tween-80 groups (*p* < 0.01) (Fig. 1C, 1D). However, linaclotide or fenchone intervention for 14 days changed the phenomenon. Compared with the IBS-C model group, the number of fecal pellets in the linaclotide, medium- and high-dose fenchone groups was obviously increased, respectively (*p* < 0.05, *p* < 0.01), and the fecal water content in the linaclotide, low-, medium-and high-dose fenchone groups was obviously enhanced, respectively (*p* < 0.05, *p* < 0.01). In terms of fecal pellet number and fecal water content, only the low-dose fenchone group among the three different doses was lower than those in the linaclotide group (*p* < 0.05), and no obvious difference exists between the medium- or high-dose fenchone and linaclotide groups.

### Effect of Fenchone on Visceral Hypersensitivity and Intestinal Transit Rate

**Fig. 2:**
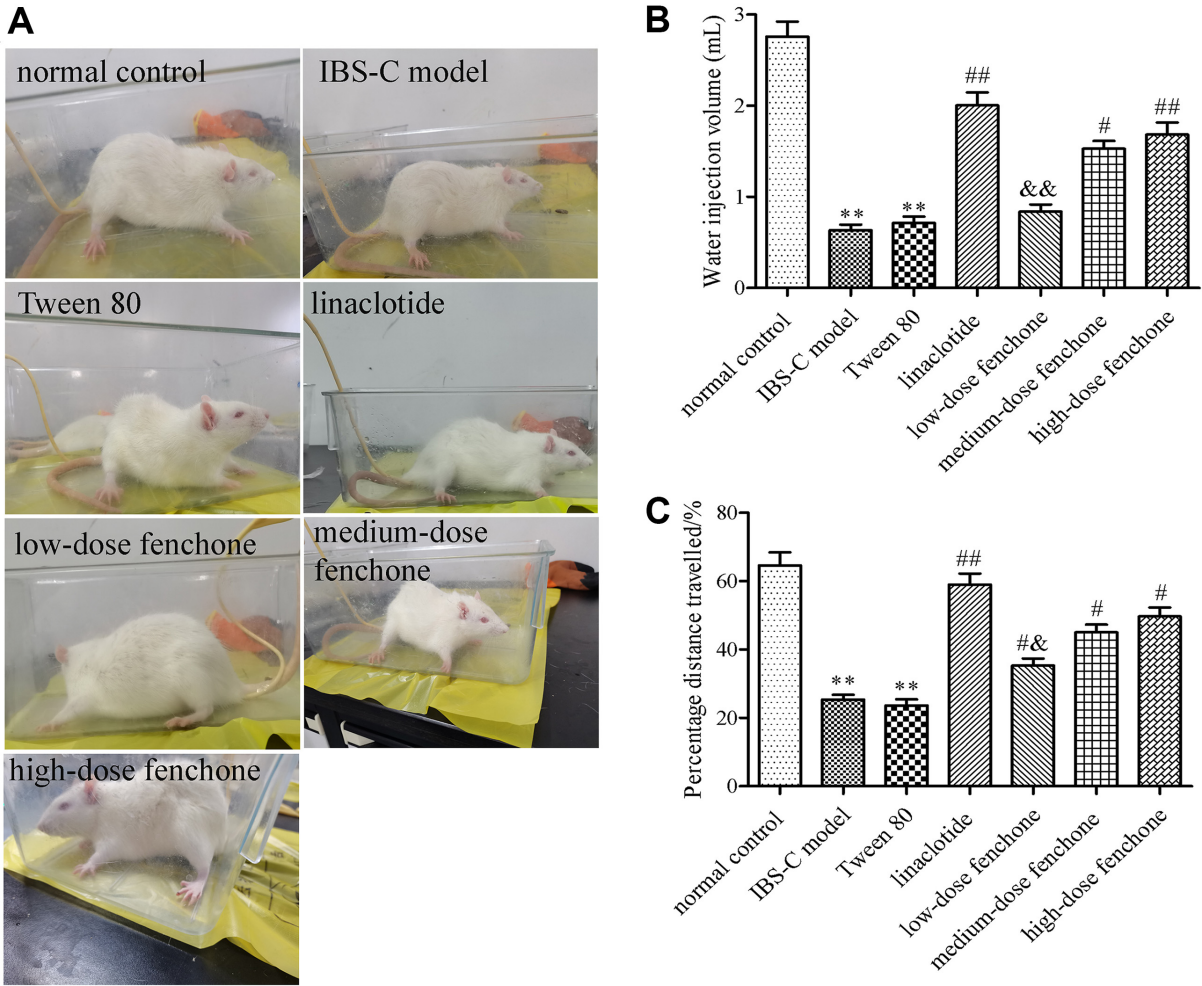
Effect of fenchone on visceral hypersensitivity and intestinal transit rate. (**A, B**) Visceral hypersensitivity. (**C**) intestinal transit rate. Data were represented as mean ± SD. **p < 0.01, compared with normal control group; ^#^*p* < 0.05, ^##^*p* < 0.01, compared with IBS-C model group; ^&^*p* < 0.05, ^&&^*p* < 0.01, compared with linaclotide group.

The water injection volume that caused the AWR to reach three points was different for each group. As shown in Fig. 2A and 2B, the IBS-C model group exhibited decreased water injection volume compared with the normal control group (*p* < 0.01). After treatment with linalotide, medium-, or high-dose fenchone, the decreased water injection volume was partially offset (*p* < 0.05, *p* < 0.01). Moreover, the water injection volume in the low-dose fenchone group was obviously lower than that in the linaclotide group (*p* < 0.01). As shown in Fig. 2C, the percentage distance traveled decreased in the IBS-C model group compared with the normal control group (*p* < 0.01). However, compared to the IBS-C model group, the intestinal transit rate in the linaclotide, low-, medium-, and high-dose fenchone groups was significantly increased (*p* < 0.05, *p* < 0.01).

### Effect of Fenchone on Gastrointestinal Hormone Levels

**Fig. 3:**
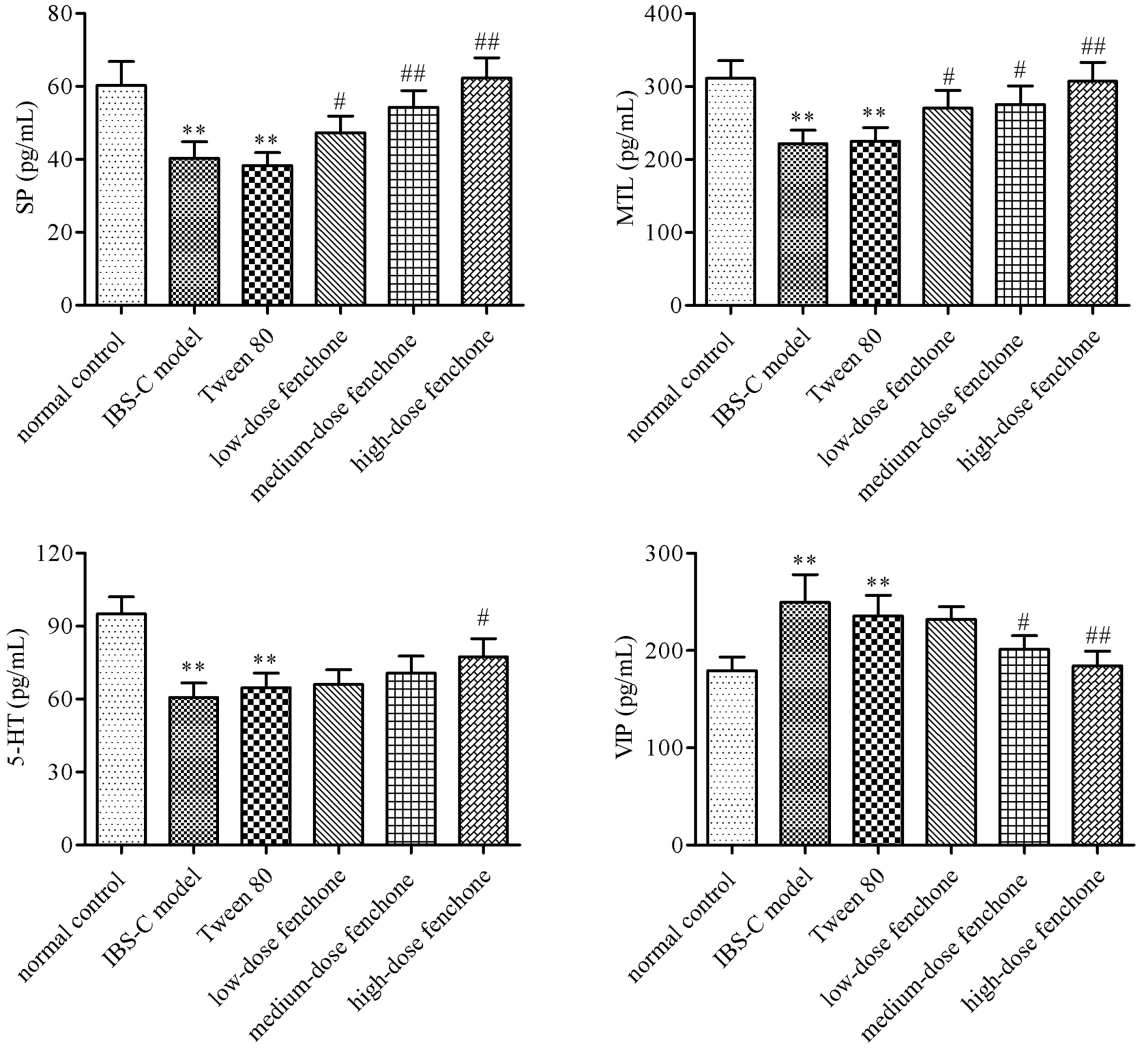
Effect of fenchone treatment on serum gastrointestinal hormone levels in IBS-C rats. (**A**) SP level. (**B**) MTL level. (**C**) 5-HT level. (**D**) VIP level. Data were represented as mean ± SD. **p < 0.01, compared with normal control group; ^#^*p* < 0.05, ^##^*p* < 0.01, compared with IBS-C model group.

To further assess the influence of fenchone on intestinal motility, gastrointestinal hormone levels were determined in every group. Fig. 3 indicated that ice water remarkably disturbed the gastrointestinal hormone levels. The levels of SP, MTL and 5-HT were decreased by 33, 29, and 36%, respectively, while the VIP level was increased by 40% in the IBS-C model group compared with the normal control group (*p* < 0.01). Low-, medium-, and high-dose fenchone enhanced the decreased SP level by 18, 36, and 55%, MTL level by 22, 24, and 38%, and 5-HT by 8, 16, and 27%, respectively. In contrast, low-, medium-, and high-dose fenchone reduced the increased VIP level by 8, 20, and 27%, respectively. There were no statistical differences in the VIP, SP, MTL and 5-HT levels between the IBS-C and Tween-80 groups in the present research.

### Effect of Fenchone on SCF/c-Kit Signaling Pathway

**Fig. 4:**
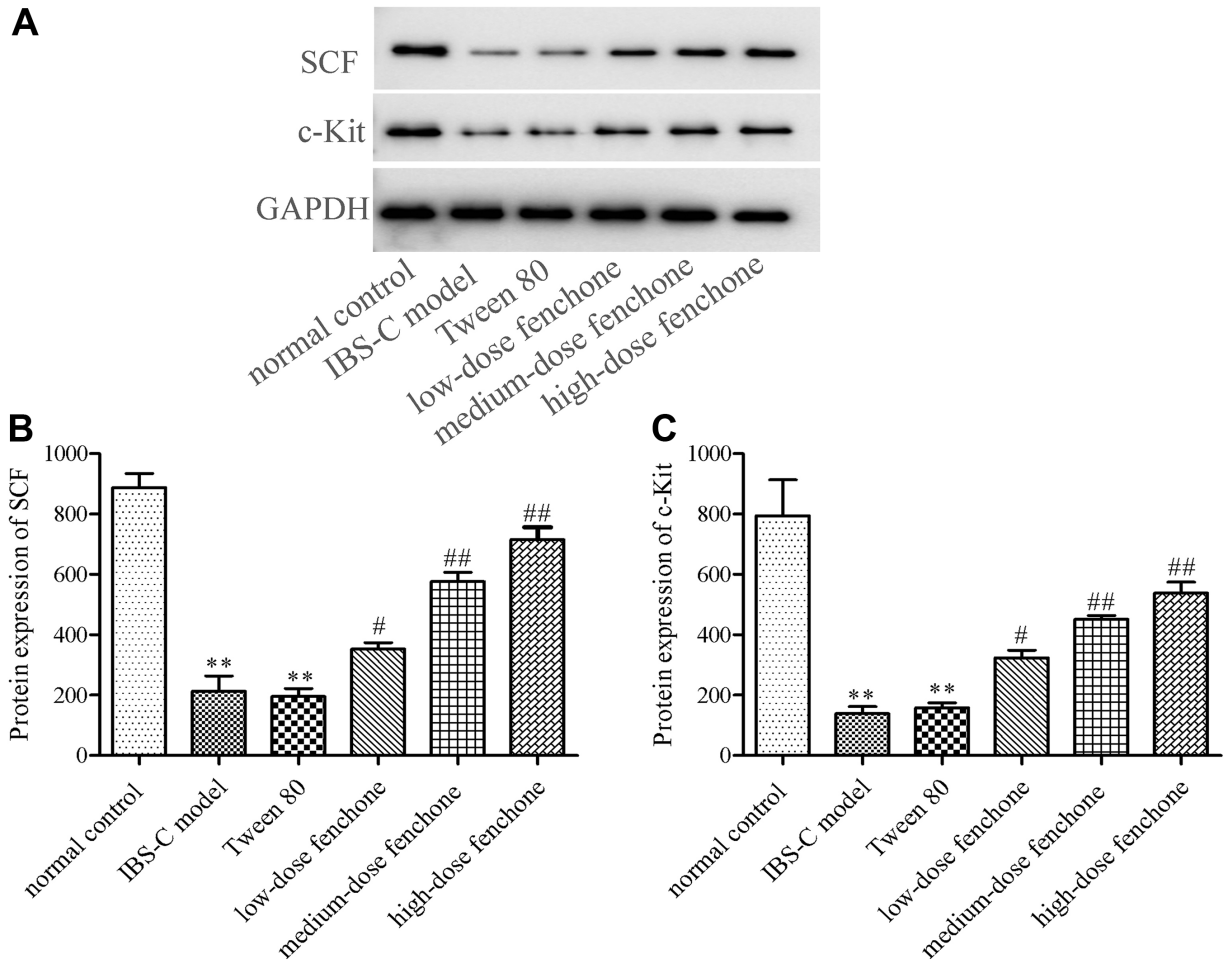
Effect of fenchone on the protein expression of SCF and c-Kit. (**A**) Western blot assay. (**B**) Quantitative analysis. Data were represented as mean ± SD. **p < 0.01, compared with normal control group; ^#^*p* < 0.05, ^##^*p* < 0.01, compared with IBS-C model group.

**Fig. 5:**
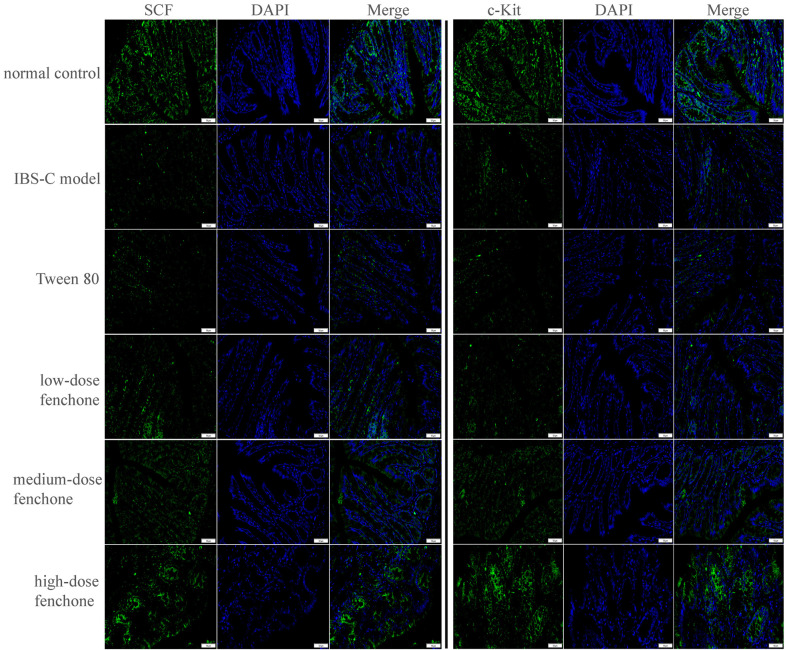
Effect of fenchone on the number of positive cells of SCF and c-Kit in colonic tissue via immunofluorescence microscopy. The tissue was immunostained with DAPI (blue) and anti-SCF-FITC or anti-c-Kit-FITC (green) (200X).

We assessed the influence of fenchone on the SCF/c-Kit signaling pathway. As shown in Fig. 4A and 4B, the protein expressions of SCF and c-Kit were significantly reduced in the IBS-C model group, compared with those of the normal control group (*p* < 0.01), whereas the two protein expressions were increased by low-, medium-, and high-dose fenchone (*p* < 0.05, *p* < 0.01). Compared with the normal control group, the number of SCF- and c-Kit-positive cells was reduced in the IBS-C model group (Fig. 5). The SCF- and c-Kit-positive cell numbers in the IBS-C model were almost the same as those in the Tween-80 group. However, the decreased numbers were reversed by fenchone, especially the high-dose fenchone.

### Fenchone Influenced the Alpha- and Beta-diversity of Gut Microbiota

**Fig. 6:**
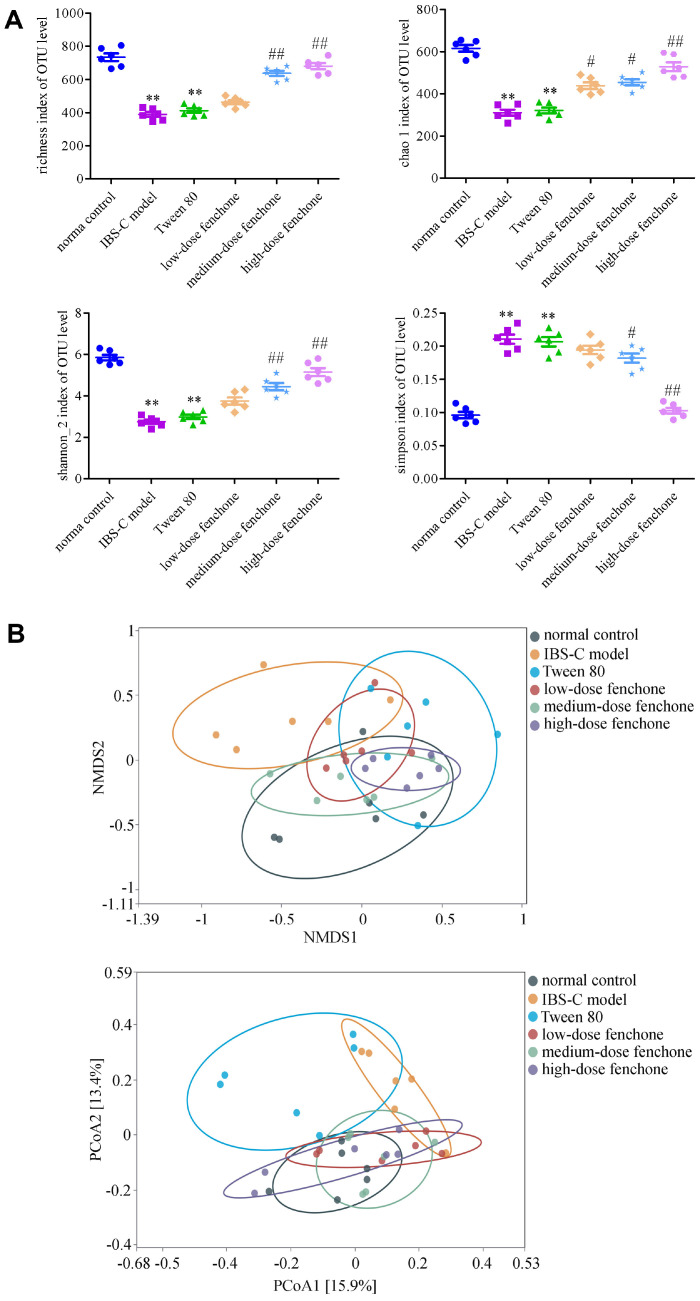
Effect of fenchone on the alpha- and beta-diversity of intestinal flora. (**A**) alpha-diversity. (**B**) beta-diversity. Data are expressed as means ± SD (*n* = 6). *p < 0.05, **p < 0.01, compared with the normal control group; ^#^*p* < 0.05, ^##^*p* < 0.01, compared with the IBS-C model group.

To explore the function of intestinal flora in IBS-C and the treatment mechanism of fenchone, 16S rRNA gene sequencing was performed. The richness, Chao1, Shannon_2, and Simpson indices could reflect a-diversity. The a-diversity of different samples is proportional to the first three indices but is negatively associated with the Simpson index. The richness and Chao1 indices represent the community's species abundance, whereas the Shannon_2 and Simpson indices refer to the community's species diversity. As shown in Fig. 6A, the richness, Chao1, and Shannon_2 indices of the IBS-C model group reduced significantly compared with those of the normal control group (*p* < 0.01). The Simpson index of the IBS-C model group increased significantly compared with that of the normal control group (*p* < 0.01). However, the richness and Shannon_2 indices of the medium-and high-dose fenchone groups, and the Chao1 index of the low-, medium-, and high-dose fenchone groups were higher than those of the IBS-C model group (*p* < 0.05, *p* < 0.01). The Simpson index of the medium- and high-dose fenchone groups were lower than that of the IBS-C model group (*p* < 0.05, *p* < 0.01). There were no significant differences in the a-diversity between the IBS-C model and Tween-80 groups. These data indicated that fenchone could improve the a-diversity of IBS-C rats. The impact of fenchone on the β-diversity of gut microbiota was determined by Principal Coordinates Analysis (PCoA) and non-metric multidimensional scaling (NMDS) analysis based on Bray-Curtis distances at the OTU level. The normal control and IBS-C model groups were obviously distinguished in the PCoA and NMDS analysis diagrams (Fig. 6B). However, the distances did not differ significantly among the normal control, low-, medium-, and high-dose fenchone groups.

### Species Community Analysis

**Fig. 7:**
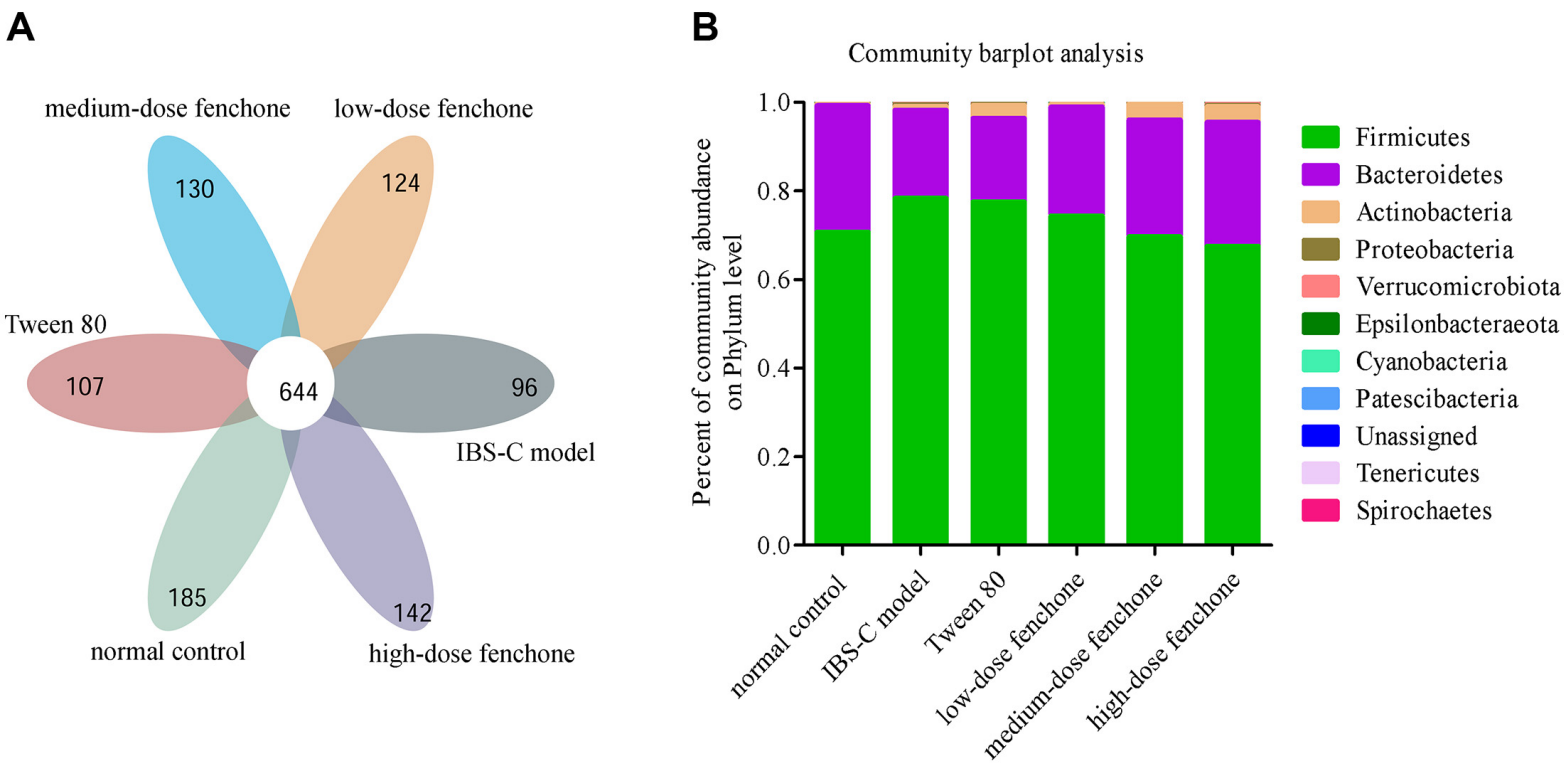
Effect of fenchone on the gut microbiota balance. (**A**) Venn diagram. (**B**) community barplot analysis on the phylum level.

**Fig. 8:**
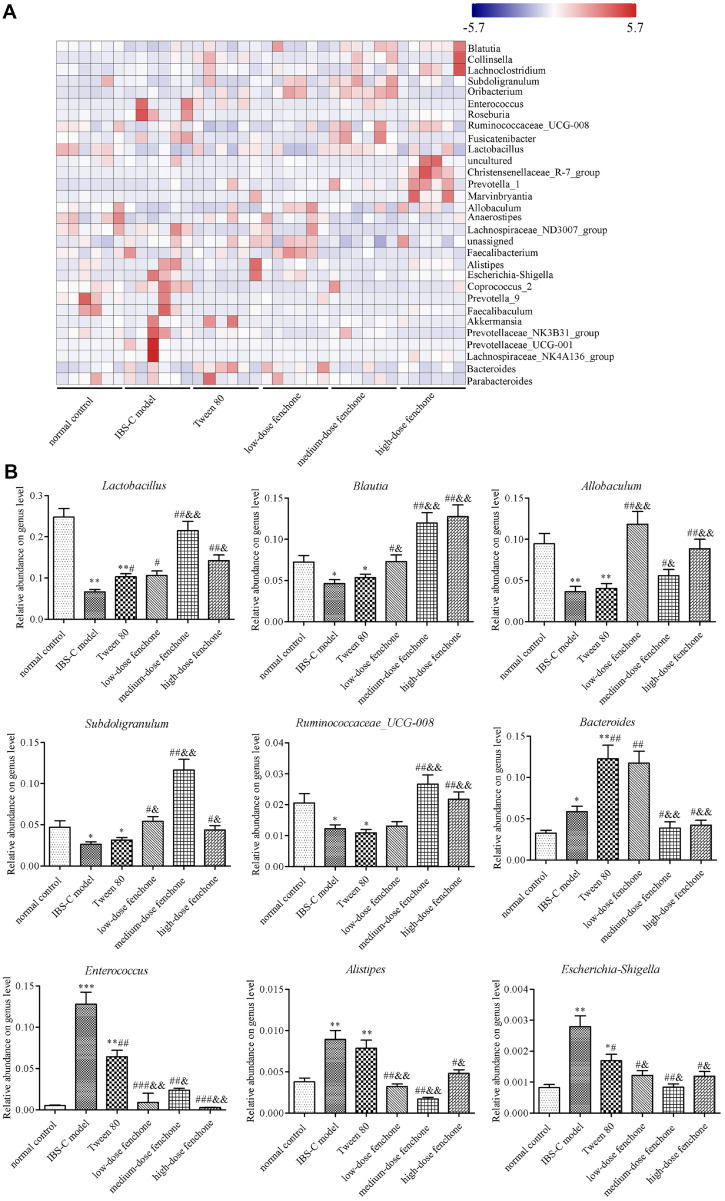
Fenchone regulated microbiota composition in IBS-C rats. (**A**) Community heatmap analysis on the genus level. (**B**) The relative abundance of *Lactobacillus*, *Blautia*, *Allobaculum*, *Subdoligranulum*, and *Ruminococcaceae_UCG-008*, *Bacteroides*, *Enterococcus*, *Alistipes*, and *Escherichia-Shigella*. Data are expressed as means ± SD (*n* = 6). *p < 0.05, **p < 0.01, ***p < 0.001, compared with the normal control group; ^#^*p* < 0.05, ^##^*p* < 0.01, #^##^*p* < 0.001, compared with the IBS-C model group; ^&^*p* < 0.05, ^&&^*p* < 0.01, compared with the Tween-80 group.

Venn diagram analysis (Fig. 7A) at OTU level identified a total of 1,428 OTUs, of which 644 were shared by every group, with 185, 96, 107, 124, 130, and 142 group-unique OTUs in the normal control, IBS-C model, Tween-80, low-, medium-, and high-dose fenchone groups, respectively. The major bacterial communities on the phylum level were Firmicutes, Bacteroidetes, and Actinobacteria (Fig.7B). The ratio of Firmicutes to Bacteroidetes was higher in IBS-C model group than that in the normal control group. However, this ratio could be reduced by fenchone treatment. Changes in the intestinal flora composition of fenchone were further analyzed using community heatmap analysis on the genus level (Fig. 8). The relative abundance of *Lactobacillus*, *Blautia*, *Allobaculum*, *Subdoligranulum*, and *Ruminococcaceae_UCG-008* was reduced in the IBS-C model and Tween-80 groups compared with that in the normal control group (*p* < 0.05, *p* < 0.01). The relative abundance of *Lactobacillus* was higher in Tween-80 group than that in the IBS-C model group (*p* < 0.05). However, there was no significant difference between IBS-C model and Tween-80 groups on the relative abundance of *Blautia*, *Allobaculum*, *Subdoligranulum*, and *Ruminococcaceae_UCG-008*. Compared with the IBS-C model group, the relative abundance of *Lactobacillus*, *Blautia*, *Allobaculum*, *Subdoligranulum*, and *Ruminococcaceae_UCG-008* was obviously enhanced in the low-, medium-, and high-dose fenchone groups (*p* < 0.05, *p* < 0.01). More importantly, compared with the Tween-80 group, the relative abundance of *Lactobacillus*, *Blautia*, *Allobaculum*, *Subdoligranulum*, and *Ruminococcaceae_UCG-008* was remarkably increased by fenchone treatment (*p* < 0.05, *p* < 0.01), except *Lactobacillus* in the low-dose fenchone. The relative abundance of *Bacteroides*, *Enterococcus*, *Alistipes*, and *Escherichia-Shigella* were increased in the IBS-C model and Tween-80 groups compared with that in the normal control group (*p* < 0.05, *p* < 0.01, *p* < 0.001). The relative abundance of *Bacteroides* was higher in Tween-80 group than that in the IBS-C model group (*p* < 0.01), and the relative abundance of *Enterococcus* and *Escherichia-Shigella* was lower in the Tween-80 group than that in the IBS-C model group (*p* < 0.05, *p* < 0.01). However, there was no significant difference between IBS-C model and Tween-80 groups on the relative abundance of *Alistipes*. Compared with the IBS-C model group, the relative abundance of *Bacteroides*, *Enterococcus*, *Alistipes*, and *Escherichia-Shigella* was obviously decreased by the fenchone intervention groups (*p* < 0.05, *p* < 0.01, *p* < 0.01), except for *Bacteroides* in the low-dose fenchone group. Additionally, compared with the Tween-80 group, the relative abundance of *Bacteroides*, *Enterococcus*, *Alistipes*, and *Escherichia-Shigella* was remarkably reduced by fenchone treatment (*p* < 0.05, *p* < 0.01), except for *Bacteroides* in the low-dose fenchone group. These results indicated that fenchone could improve gut microbiota composition, promoting a healthier intestinal microbiota, similar to that of the normal control group.

## Discussion

In this study, intragastric administration of cold water (0–4°C) once a day for 14 consecutive days in SD rats contributed to the establishment of the IBS-C model. This model was described as reduced body weight, number of fecal pellets, fecal moisture, and intestinal transit rate. Elevated visceral hypersensitivity was also observed via the AWR following colorectal distention. Previous researchers also reported the visceral hypersensitivity in the IBS-C model caused by intragastric administration of cold water (1, 12, 25). Linaclotide is a guanylate cyclase-C agonist, which could promote intestinal secretion and decrease the visceral nociception threshold. Importantly, linaclotide has been approved by the US FDA and EU Medicines Agency for alleviation of IBS-C. Therefore, we used linaclotide as a positive drug to observe the effect of fenchone on IBS-C. Our results indicated that fenchone could increase the body weight, number of fecal pellets, fecal moisture, and intestinal transit rate, while decreasing visceral hypersensitivity.

Gastrointestinal motility dysfunction always brings with it visceral hypersensitivity in IBS-C patients, which is a strong response to stimuli and possibly caused by alterations in the processing of afferent signals from visceral neurons [12]. In addition, 5-HT stimulates enteric neurons and vagus nerve and promotes gastrointestinal motility, which directly induces the contraction of colon smooth muscle by promoting choline expression pathway [27]. The 5-HT content plays a key role in gastrointestinal function and fecal excretion [28]. Our results indicated that fenchone increased 5-HT content in serum to ameliorate the constipation symptom of IBS-C rats. The intestinal nervous system is made up of various neurotransmitters, including inhibitory and excitatory neurotransmitters, and the enteric neurotransmitters have attracted considerable attention from researchers [29]. Colonic receptors pick up their corresponding neurotransmitters and then transfer them to neighboring smooth muscle cells, which accelerate bowel motility [30]. In this study, fenchone increased the serum contents of excitatory neurotransmitters (SP, MTL, and 5-HT) and decreased the serum content of inhibitory neurotransmitters (VIP) of the IBS-C model in rats. Moreover, compared with the IBS-C model group, the intestinal transit rate was significantly increased in fenchone treatment groups. Therefore, fenchone enhanced intestinal movement, perhaps by modulating the balance between excitatory and inhibitory neurotransmitters.

Reduced intestinal movement results in constipation. ICC is characterized by the pacemaker cells of the digestive tract and possesses the function of regulating gastrointestinal peristalsis [31]. ICC is also an important participant in promoting gastrointestinal movement by receiving neurotransmitters through receptors. Kiwi berry extracts could strengthen GI transport performance by increasing the protein level of SCF and c-Kit [32]. Konjac oligosaccharides and konjac glucomannan also activated the SCF/c-Kit pathway, and thus promoting GI motility [33]. In this study, both western blot and immunofluorescence experiments confirmed that fenchone could promote the protein level of SCF and c-Kit. Consequently, fenchone increased the GI transit rate compared with the IBS-C model group.

Variations in intestinal flora composition are closely related to IBS-C. Probiotic intervention has positive influence on IBS-C patients. *Lactobacillus* is usually reduced in IBS-C patients compared with healthy controls [34]. Shang *et al*. found that the increase in fecal *Lactobacillus* may improve the imbalance in the intestinal flora and help maintain normal GI function [35]. Also, the occurrence and development of IBS-C are closely associated with *Blautia*. The abundance of *Blautia* in the intestinal tract is beneficial for health. MRx1234 is a live biotherapeutic product containing a strain of *Blautia hydrogenotrophic*. The bowel habits and abdominal pain in patients with IBS who received MRx1234 were significantly improved compared with those of patients who received a placebo [36]. Moreover, *Allobaculum*, *Subdoligranulum*, and Ruminococcaceae are important butyrate-producing bacteria that keep the intestinal microenvironment acidic [37]. Butyrate provided energy to the colonocyte and facilitated tight-junction protein assembly via multiple pathways [38]. *Allobaculum* is also considered to take part in inflammatory processes and enhance the host intestinal immune response [39]. In this research, the relative abundance of *Lactobacillus*, *Blautia*, *Allobaculum*, *Subdoligranulum*, and *Ruminococcaceae_UCG-008* was increased in fenchone groups compared with the IBS-C group. *Bacteroides* and *Enterococcus* are an important pathogen that causes intestinal mucosal damage and immune defense dysfunction [40, 41]. Ji *et al*. found that the more serious the intestinal injury, the higher the level of *Enterococcus* [42]. Some other researchers reported that there was no obvious difference between IBS-C and healthy participants [43]. Additionally, the abundance of *Enterococcus* and *Escherichia-Shigella* increased with the severity of inflammatory response, thereby serving as a biomarker of inflammation [44, 45]. Our results indicated that fenchone could reverse the increased abundance of *Enterococcus* and *Escherichia-Shigella* in the IBS-C group. *Alistipes*, anaerobic bacteria, principally exist in the gut microbiome of healthy people. Furthermore, *Alistipes* have unique functional properties compared to other members of the phylum Bacteroidetes. The ecological imbalance of Alistipses could be beneficial or harmful. It was reported that *Alistipes* had protective function against diseases, such as colitis and cardiovascular diseases. But other researchers have shown *Alistipes* to be pathogenic in colorectal cancer and linked to mental health [46, 47]. In our study, we found that the relative abundances of *Bacteroides*, *Enterococcus*, *Alistipes*, and *Escherichia-Shigella* were enhanced in the IBS-C group compared with those in the normal control group, but the abundance of these bacteria was greatly reduced after fenchone therapy.

## Conclusion

In conclusion, fenchone could improve IBS-C symptoms, including body weight, number of fecal pellets, fecal moisture, intestinal transit rate, and visceral hypersensitivity. Additionally, the potential mechanisms of fenchone alleviating IBS-C are associated with the regulation of excitatory and inhibitory neurotransmitters, SCF/c-Kit pathway, and composition of intestinal flora.
